# Magnetic Resonance Image Findings and Potential Anatomic Risk Factors for Chodromalacia in Children and Adolescents Suffering from Non-Overload Atraumatic Knee Pain in the Ambulant Setting

**DOI:** 10.3390/tomography10020019

**Published:** 2024-02-11

**Authors:** Wolf Bäumler, Daniel Popp, Patrick Ostheim, Marco Dollinger, Karin Senk, Johannes Weber, Christian Stroszczynski, Jan Schaible

**Affiliations:** 1 Department of Radiology, University Hospital Regensburg, 93053 Regensburg, Germany; marco.dollinger@ukr.de (M.D.); karin.senk@ukr.de (K.S.); christian.stros@ukr.de (C.S.); jan.schaible@ukr.de (J.S.); 2 Department of Trauma Surgery, University Hospital Regensburg, 93053 Regensburg, Germany; daniel.popp@ukr.de (D.P.); johannes1.weber@ukr.de (J.W.); 3 Bundeswehr Institute of Radiobiology, University of Ulm, 80937 Munich, Germany; patrickostheim@gmx.de

**Keywords:** MRI, knee pain, atraumatic, non overload, children, adolescents, chondromalacia patellae

## Abstract

Purpose: To evaluate magnetic resonance image (MRI) findings in children and adolescents suffering from knee pain without traumatic or physical overload history and to identify potential anatomic risk factors. Material and Methods: A total of 507 MRIs of 6- to 20-year-old patients (251 males; 256 females) were evaluated with regard to detectable pathologies of the knee. The results were compared to a control group without pain (*n* = 73; 34 males; 39 females). A binary logistic regression model and t-tests for paired and unpaired samples were used to identify possible risk factors and significant anatomic differences of the study population. Results: In 348 patients (68.6%), at least one pathology was detected. The most commonly detected finding was chondromalacia of the patellofemoral (PF) joint (n = 205; 40.4%). Chondral lesions of the PF joint occurred significantly more often in knee pain patients than in the control group (40% vs. 11.0%; *p* = 0.001), especially in cases of a patella tilt angle > 5° (*p* ≤ 0.001), a bony sulcus angle > 150° (*p* = 0.002), a cartilaginous sulcus angle > 150° (*p* = 0.012), a lateral trochlear inclination < 11° (*p* ≤ 0.001), a lateralised patella (*p* = 0.023) and a Wiberg type II or III patella shape (*p* = 0.019). Moreover, a larger patella tilt angle (*p* = 0.021), a greater bony sulcus angle (*p* = 0.042), a larger cartilaginous sulcus angle (*p* = 0.038) and a lower value of the lateral trochlear inclination (*p* = 0.014) were detected in knee pain patients compared to the reference group. Conclusion: Chondromalacia of the PF joint is frequently observed in children and adolescents suffering from non-overload atraumatic knee pain, whereby a patella tilt angle > 5°, a bony sulcus angle > 150°, a cartilaginous sulcus angle > 150°, a lateral trochlear inclination < 11°, a lateralised patella and a Wiberg type II or III patella shape seem to represent anatomic risk factors.

## 1. Introduction

Knee pain is considered to be one of the most common musculoskeletal afflictions in young patients [1,2]. Particularly in children and adolescents, apart from traumatic causes, atraumatic causes play a part in the genesis of knee pain. These causes include, in particular, physical overload, whose potential negative effects on the immature skeleton have already been described in the literature [1,3,4]. Nevertheless, knee pain is also often reported by children or adolescents without a recent traumatic injury or physical overload. To evaluate the reason for the knee pain in this patient group, a detailed anamnesis followed by a thorough physical examination is essential. Unfortunately, these examination methods are sometimes not sufficient for analysing the reason for the complaints, making further investigations necessary. Currently, magnetic resonance imaging (MRI) is the preferred imaging technique to evaluate the knee joint [5,6]. Compared to other techniques like computed tomography or X-ray, MRI can be applied without ionising radiation, making it the method of choice, especially in young patients [7]. Nowadays, MRI is widely available, and the average examination time of 10–12 min is quite tolerable. Furthermore, MRI is considered the most eligible imaging method for assessing soft tissue, including articular cartilage and ligaments [2]. Besides offering the opportunity to directly diagnose knee diseases, MRI can visualise potential anatomic or physiological risk factors, e.g., a pathological sulcus angle, which may cause further symptoms [8,9]. Although knee pain has been a frequently studied topic in the last few years, the reason for knee pain in children and adolescents without traumatic or physical overload history often remains unknown. The aim of the current study was to evaluate MRI findings of children and adolescents in outpatient treatment suffering from non-overload atraumatic knee pain and to identify potential anatomic risk factors, which may affect these complaints.

## 2. Material and Methods

### 2.1. Study Design, Participant Selection and Patient Characteristics

The retrospective observational study was approved by the local Ethics Committee (approval number 23-3486-104). The study was conducted in accordance with the relevant guidelines and regulations. To investigate the MRI findings and potential anatomic risk factors of children and adolescents suffering from non-overload atraumatic knee pain, the authors analysed all magnetic resonance tomographies performed between December 2021 and September 2023 in patients between 6 to 20 years of age in a radiological practice in the authors’ home country. In the current study, the following inclusion criteria were applied: (I) a physical examination of the affected knee was conducted within the last 3 months followed by a referral for MRI by an orthopaedic surgeon. (II) Absence of traumatic or physical overload history for the affected knee. (III) Absence of surgery on the afflicted knee. (IV) Written informed consent was obtained from the patient for the acquisition of MRI and the anonymous use of the data for scientific purposes.

In total, 507 patients fulfilled the inclusion criteria. Mean age was 16.1 ± 3.2 years (range: 6–20 years). The study population consisted of 251 males (49.5%) and 256 females (50.5%). In 236 cases (46.5%), the right knee was affected. A total of 271 patients (53.5%) suffered from left knee pain. A total of 250 patients (49.3%) suffered from knee pain of the dominant limb.

The results were compared to a reference group consisting of 73 study participants of similar age (mean age: 18.2 ± 2.9 years; range: 8–20) without knee pain. In all, 34 participants were males (46.6%), 39 were females (53.4%). In 31 (42.5%) cases, the right knee was examined; in 42 cases (57.5%), the MRI of the left knee was evaluated. In 35 participants of the control group (47.9%), the knee of the dominant limb was examined. For the control group, the above-mentioned inclusion criteria II-IV were applied.

### 2.2. Image Acquisition and Evaluation

All MRIs were performed using a 1.5 Tesla MR scanner (MAGNETOM Altea, Siemens Healthcare GmbH, Erlangen, Germany). For each examination, the same MR coil was used. To acquire imaging data digitally, a picture archiving and communication system (PACS) was used. In all patients, a standardised testing protocol was applied. The patients were positioned with 15–20 degrees of knee flexion. The testing protocol included sagittal fat-saturated proton density-weighted turbo spin echo (PD-tse-fs) images [repetition time (TR)/echo time (TE) 3480/35 ms; 400 × 260 matrix]. Moreover, it contained sagittal T1-tse (TR/TE 699/10 ms; 400 × 260 matrix), coronal PD-tse-fs (TR/TE 3120/37 ms; 448 × 336 matrix) and axial PD-tse-fs (TR/TE 3600/37 ms; 448 × 314 matrix) images. The standardised field of view was set to 180 mm, the flip angle to 180° and the thickness to 3 mm.

**Figure 1:**
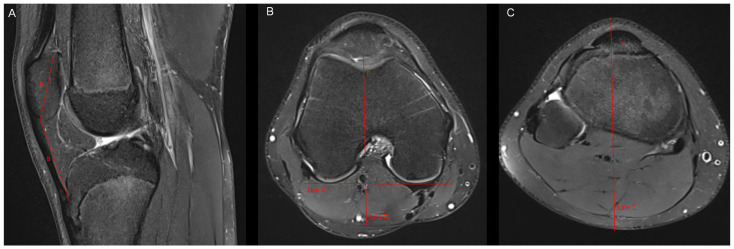
Measurement of the Insall–Salvati ratio and the tibial tubercle deviation. (**A**) Insall–Salvati ratio: Measurement of the largest superiorinferior diameter of the patella (A) in sagittal MR-images in which the tibial insertion of the patellar tendon can be identified. Measurement of the patellar tendon length (B). Insall–Salvati ratio = B/A. (**B**,**C**) Tibial tubercle deviation: We drew a line (line X) that connected the posterior aspect of both femur condyles in the MR-image of the nearest subchondral spot of the femoral trochlea. After that, we drew a perpendicular line to line X (line Z), which passed the nearest subchondral spot of the femoral trochlea (P). We scrolled line Z to the axial level of the tibial insertion of the patellar tendon. The tibial tubercle deviation represents the distance between line Z and the midpoint of the insertion of the patellar tendon at the tibial tubercle (T).

**Figure 2:**
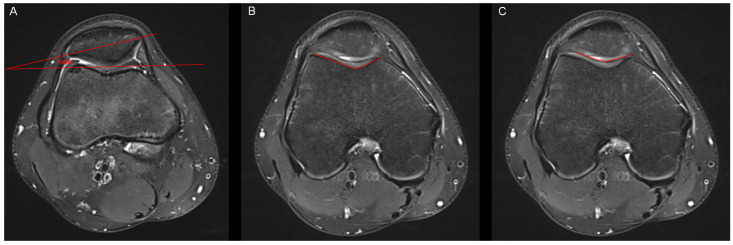
Measurement of the patella tilt angle, the bony sulcus angle and the cartilaginous sulcus angle. (**A**) In accordance with Grelsamer et al. [10], the patella tilt angle (alpha) was defined as the subtended by a line joining the medial and lateral edges of the patella and a horizontal line measured in the axial slice with the largest mediolateral diameter of the patella. (**B**) The bony sulcus angle was defined as the angle between the bony medial and lateral facets. (**C**) The cartilaginous sulcus angle was defined as the angle between the cartilaginous medial and lateral facets.

**Figure 3:**
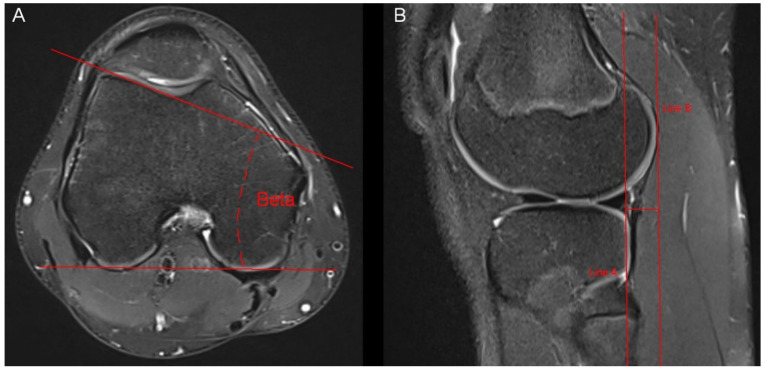
Measurement of the lateral trochlear inclination and the anterior tibial translocation. (**A**) The lateral trochlear inclination was measured through an angle (beta) between two lines. The first line was connected the posterior aspect of both femur condyles. The second line was placed on the lateral facet of the trochlea. Measurement was conducted in the axial slice offering the largest distance of the lateral facet of the trochlea. (**B**) The anterior tibial translocation was measured in sagittal MR-images showing the lateral femur condyle. We drew two parallel lines (line A and line B), which were also parallel to the axis of the tibia. The distance between the two lines represents the anterior tibial translocation.

All MR images were analysed independently by two radiologists with 9 years and 10 years of experience and a special certification in musculoskeletal imaging, respectively. After evaluating the MR images, the two radiologists compared their results. In cases of discrepancy, a consensus decision was taken after discussing the case in detail. The radiologists assessed the patellofemoral (PF) joint, the tibiofemoral (TF) joint and the extensor mechanism of the affected knee. Furthermore, parameters representing potential anatomic risk factors for the development of prospective knee complaints were evaluated. The MRI assessment included abnormalities in the cruciate and collateral ligaments, lateral and medial meniscus, PF and TF cartilage (modified Outerbridge-classification; Grad I: Inhomogeneous areas of the cartilage without substantial defect; Grade II: substantial defect up to 50% of the cartilage height; Grade III: substantial defect > 50% of the cartilage height without total focal loss of cartilage; Grade IV: extensive area with total loss of cartilage), the extensor mechanism (quadriceps tendinopathy, patellar tendinopathy, praepatellar/infrapatellar Bursitis, Osgood Schlatter disease), the presence and thickening of suprapatellar/infrapatellar/medial patellar/lateral plica, bony lesions, Baker cyst, inflammatory changes of the Hoffa fat body (Hoffaitis), articular effusion and other intra-articular abnormalities such as ganglions or loose joint bodies. Chondromalacia was defined as inhomogeneity, including fibrillations or swelling without substantial defect or proven substantial defect of the cartilage. Chondromalacia was only diagnosed if artificial changes of the cartilage could definitely be excluded by the observer. If the cartilage could not be evaluated without restrictions because of artefacts, the MRI sequence was acquired again. In cases of doubt or suspicion on artificial changes of the cartilage, the patient was not included in the current study to avoid misdiagnosis. Thickened plicas were defined as > 2 mm. An articular effusion was diagnosed if the level of the intraarticular effusion exceeded the highest point of the patella in the sagittal PD-tse-fs images. All effusions showing lower levels were defined as physiological. For the assessment of potential anatomic risk factors, several anatomic characteristics were evaluated. The Insall–Salvati ratio (patella baja < 0.8; patella alta > 1.2) was observed, as the vertical position of the patella is well described via this method. By studying the presence of a lateralised patella, the tibial tubercle deviation (distance from the tibial tubercle to the trochlear groove; abnormal > 15 mm) and the patella tilt angle (abnormal > 5°), the horizontal and anterior–posterior positions could be defined. To evaluate the anatomical interaction of the patella and its femoral joint partner, the bony and cartilaginous sulcus angles (in both cases: abnormal > 150°) and the lateral trochlear inclination (abnormal < 11°) were regarded closely. As the configuration of the patella may vary obviously, the patella shape (Wiberg classification) was also regarded as a potential risk factor. Furthermore, the femorotibial interaction was assessed by measuring the anterior tibial translocation (abnormal > 7 mm). Figure 1, Figure 2 and Figure 3 provide an overview of the different measurement methods. The mentioned thresholds were set based on the current literature.

### 2.3. Statistical Analysis

The normal distribution of all data was reviewed graphically and by using the Shapiro–Wilk test. All collected data are presented as frequency counts and percentages. To identify potential risk factors for the occurrence of non-overload atraumatic knee pain in children and adolescents, a binary logistic regression model was used. Moreover, a *t*-test for paired samples was utilised to evaluate significant differences in the prevalent anatomical conditions of subgroups of the study population. To compare the results of the study population and the control group, a *t*-test for unpaired samples was used. As effect estimates, 95% confidence intervals are presented. A *p*-value of ≤0.05 was considered statistically significant in all statistical analyses. All statistical analyses were performed with SPSS statistic (IBM SPSS Statistics, version 28, IBM, Armonk, New York, USA).

## 3. Results

A normal distribution was proven for all data. In 159 patients (31.4%), no pathological finding could be proven. In 348 patients (68.6%), at least one pathology was detected in the performed MRI. The most frequent disease was the chondral lesion of the PF joint (n = 205; 40.4%) whereby chondromalacia patellae Grade I was observed in 60 (11.8%), Grade II in 74 (14.6%), Grade III in 67 (13.2%), and Grade IV in 4 patients (0.8%). The second and third most commonly detected findings were articular effusion (n = 123; 24.3%) and bone oedema (n = 48; 9.5%). Both pathologies were mostly associated with another pathological finding. Only in four patients (0.8%) did articular effusion represent a solitary finding. Bone oedema was proven as the sole change in two cases (0.4%). Moreover, Baker’s cyst (n = 21; 4.1%), thickening of Plica mediopatellaris (n = 15; 3.0%), Morbus Osgood Schlatter (n = 14; 2.8%), patellar tendinopathy (n = 14; 2.8%) and mucoid degeneration of the medial meniscus (n = 12; 2.4%) were observed in some small subgroups of the study population. Table 1 summarises all pathological findings of both groups. Table 2 presents the assessment of the anatomical situation of the knee pain group. Patella tilt angle > 5° (n = 199; 39.3%), patella alta (n = 125; 24.7%), lateral trochlear inclination < 11° (n = 125; 24.7%), cartilaginous sulcus angle > 150° (n = 120; 23.7%), bony sulcus angle > 150° (n = 101; 19.9%) and patellar lateralisation (n = 97; 19.1%) represented frequent anatomical variants. The anatomical parameters of the control group are summarised in Table 3. Wiberg type I was the most frequently described patella shape in both groups, as shown in Table 4. With chondral lesions of the PF joint being the most commonly detected findings in the knee pain group, potential associated anatomical risk factors were assessed. In cases of a patella tilt angle > 5° (*p* =< 0.001), a bony sulcus angle > 150° (*p* = 0.002), a cartilaginous sulcus angle > 150° (*p* = 0.012), a lateral trochlear inclination < 11° (*p* =< 0.001), a lateralised patella (*p* = 0.023) and a Wiberg type II or III patella shape (*p* = 0.019) chondral lesions of the PF joint were proven significantly more often in the knee pain group (Table 5). Furthermore, in the knee pain group, significant differences between patients with and without patellofemoral chondral lesions were detected concerning the patella tilt angle (*p* = 0.002), the bony sulcus angle (*p* = 0.017), the cartilaginous sulcus angle (*p* = 0.007) and the lateral trochlear inclination (*p* =< 0.001). The results are presented in Table 6. Comparing the occurrence of PF chondral lesions in the knee pain group (n = 205; 40.4%) to the control group (n = 8; 11.0%), a PF cartilage lesion could be proven significantly more often in the knee pain group (*p* = 0.001). Moreover, the study demonstrates some significant anatomical differences between the two groups. A significant larger patella tilt angle was proven in the knee pain group (*p* = 0.021). Furthermore, bony and cartilaginous sulcus angles showed significantly lower values in the control group (bony sulcus angle: *p* = 0.042; cartilaginous sulcus angle: *p* = 0.038). In addition, significantly lower values of the lateral trochlear inclination were detected in the knee pain group (*p* = 0.014). Table 7 summarises the mentioned results.

## 4. Discussion

The current study suggests that chondral lesions of the PF joint are common findings in children and adolescents suffering from knee pain without traumatic or physical overload history. We also found indications that a patella tilt angle > 5°, a bony sulcus angle > 150°, a cartilaginous sulcus angle > 150°, a lateral trochlear inclination < 11°, a lateralised patella and a Wiberg type II or III patella shape seem to represent anatomic risk factors.

Knee pain is frequently observed in children and adolescents [11], whereby there is some evidence that this might often be caused by physiological or anatomical variants of the PF joint [12]. Although many reports have been published on the topic of knee pain, only little is known about non-overload atraumatic knee pain in young patients. To our knowledge, this is the first larger study that systematically addresses knee pain without traumatic or physical overload history in children and adolescents simultaneously, offering a comparison to a reference group being free of complaints.

In 40.4% of the knee pain patients, chondromalacia patellae could be proven, whereas a significantly lower number of chondromalacia patellae was detected in the control group (n = 11.0%). In a previous trial, Zhang et al. compared 354 students from a gymnastic department to 429 students from a nongymnastic department concerning the prevalence of chondromalacia patella. The authors described a prevalence of the chondral lesions of the PF joint in up to 20.1% in the gymnastic group, while in the nongymnastic group, chondral lesions of the PF joint could be observed in up to 5.6% of the examined students [13]. Another study conducted by Pihlajamäki et al. evaluated the presence of chondromalacia patellae in 56 young soldiers (median age: 19.5 years) suffering from prolonged knee pain after a longer period of physical activity. The authors reported a prevalence of chondromalacia patellae in 45% of the study population, proven by arthroscopy [14]. The findings of Zhang [13] and Pihlajamäki [14] support the widespread assumption that chondral lesions of the PF joint are often associated with physical overload. The results of the current study cannot be aligned with this thesis, as physical overload was set as an exclusion criterion, both in knee pain patients and in the control group. Instead, the present investigation illustrates that chondromalacia patellae seem to be a non-negligible potential cause, which should be clarified in children and adolescents suffering from knee pain without traumatic or physical overload history.

By evaluating possible influencing factors for chondral lesions of the PF joint in young knee pain patients, several potential risk factors were detected. A patella tilt angle > 5° was proven significantly more often in patients with PF chondral lesions (binary logistic regression model: *p* =< 0.001; *t*-test for paired samples: *p* = 0.002). Moreover, the patella tilt angle was significantly larger in the knee pain group than in the control group (*p* = 0.021). These findings support the presumption that the presence of a patella tilt might be a risk factor for the development of chondromalacia patellae. This outcome is in line with the results of Kim et al., who identified a patellar lateral tilt as a risk factor for PF instability in children [15].

An increased sulcus angle seems to represent another influencing anatomic variant. Both a bony and a cartilaginous sulcus angle > 150° were reported more frequently in anguished patients with chondromalacia patellae (bony sulcus angle: binary logistic regression model: *p* = 0.002; *t*-test for paired samples: *p* = 0.017; cartilaginous sulcus angle: binary logistic regression model: *p* = 0.012; *t*-test for paired samples: *p* = 0.007). Moreover, bony and cartilaginous sulcus angles showed significantly higher values in knee pain patients compared to the reference group (bony sulcus angle: *p* = 0.042; cartilaginous sulcus angle: *p* = 0.038). According to the literature, a sulcus angle > 150° has been defined as representative for trochlear dysplasia [12], whereby trochlear dysplasia can be observed in up to 96% of patellar dislocation events in children and adolescents [2]. Furthermore, Fones et al. concluded that an increased sulcus angle can be associated with osteochondral damage in patients with patellar instability [16]. Considering all these findings, an increased sulcus angle seems to be a serious risk factor for several knee injuries, probably including chondral lesions of the PF joint.

Another anatomic finding associated with the occurrence of chondromalacia patellae in knee pain patients was a lateral trochlear inclination < 11°. Both in the binary logistic regression model (*p* =< 0.001) and in the *t*-test for paired samples (*p* =< 0.001), significant results could be detected. Furthermore, significantly lower values of the lateral trochlear inclination were assessed in knee pain patients (*p* = 0.014) after comparing both groups. The results coincide with the outcome of Duran et al., who compared the lateral trochlear inclination of adult patients with chondromalacia patellae and a control group without cartilage lesions. The authors observed significantly lower values of lateral trochlear inclination in the chondromalacia patellae patients than in the reference group [17]. An almost identical outcome was reported by Dursun et al., who also evaluated the lateral trochlear inclination in adult patients with and without cartilage lesions in the PF joint. The investigators of this study described a significantly lower lateral trochlear inclination in patients with chondromalacia patellae, too [18]. Unlike the present investigation, the study population of these trials consisted of adult patients. However, there is some evidence in the current literature that the lateral trochlear inclination may influence the occurrence of knee complaints in children, too. As an example, a study of Djuricic et al. can be mentioned. The authors ascertained that lateral trochlear inclination may represent a risk factor for knee injuries of physically active adolescents depending on the current patella type [6]. Taking all these findings into consideration, lateral trochlear inclination seems to represent a non-negligible influencing factor for knee injuries in young patients, including chondromalacia patellae.

Another important aspect of the current study was the fact that a lateralised patella was significantly more often observed in anguished patients with chondromalacia patellae (*p* = 0.023). The lateralisation of the patella can be assigned to a spectrum of diseases, which is often termed patellofemoral instability, ranging from mild maltracking to complete lateral patellar dislocation [15]. Episodes of lateral patellar dislocation are often observed in young patients, with the first patellar dislocation generally occurring between 15 and 19 years [19,20]. The risk of progressive cartilage damage after an initial patellar dislocation is six times higher than before [21], emphasising the importance of the central position of the patellar in its joint. Although patellar lateralisation cannot be equated with a complete lateral patellar dislocation, the present trial indicates that this decentralised position of the patella might provoke chondromalacia patellae in young patients suffering from knee pain.

Last but not least, the current investigation suggests that the shape of the patella may influence the development of patellofemoral chondropathy in young knee pain patients. In patients with Wiberg type II or III, chondromalacia patellae were proven to be significantly more often than in patients without cartilage lesions (*p* = 0.019). In the literature, there is some evidence that the combination of a certain patella shape and other individual anatomic characteristics, e.g., lateral trochlear inclination, may influence the occurrence of knee injuries in young patients [6]. Aside from the potential influence of lateral trochlear inclination, which has already been discussed in this context, our study indicates that young anguished patients with Wiberg type II or III patella seem to be at a greater risk from PF chondral lesions than patients with Wiberg type I patella, regardless of the further anatomic conditions that additionally exist in these patients.

The present study has two limitations. The first is the retrospective nature of the study. The second limitation is the inhomogeneity of the study population and the control group concerning the number of participants. A comparison of the results to a reference group consisting of a larger number of participants might have increased the predictive significance of the current trial.

## 5. Conclusions

The current study suggests that chondromalacia patellae is common in children and adolescents suffering from non-overload atraumatic knee pain. Furthermore, a patella tilt angle > 5°, a bony sulcus angle > 150°, a cartilaginous sulcus angle > 150°, a lateral trochlear inclination < 11°, a lateralised patella and a Wiberg type II or III patella shape seem to represent anatomic risk factors for the development of patellofemoral chondropathy. Consequently, the trial indicates that in young patients suffering from knee pain without a traumatic or physical overload history, thorough MRI assessment is indispensable, whereby particular attention should be paid to the PF joint and its anatomic situation. The current results may be seen as a basis for further investigations. Nevertheless, additional studies optimally consisting of a larger group of patients and an appropriate reference group should be conducted in order to increase the knowledge on that topic and to detect further potential influencing factors.

## Figures and Tables

**Table 1:** Pathological findings of patients with knee pain and the control group.

Pathological Findings	Knee Pain Group		Control Group	
	Number of Patients	%	Number of Patients	%
Bone oedema	48	9.5	3	4.1
Distorsion of the medial collateral ligament	5	1.0	0	0
Mucoid degeneration of the medial meniscus	12	2.4	4	5.5
Medial meniscal tear	2	0.4	0	0
Mucoid degeneration of the lateral meniscus	4	0.8	2	2.7
Lateral meniscal tear	2	0.4	0	0
Chondral lesion in the TF joint				
Grade I	7	1.4	3	4.1
Grade II	3	0.6	2	2.7
Chondral lesion in the PF joint				
Grade I	60	11.8	4	5.5
Grade II	74	14.6	3	4.1
Grade III	67	13.2	1	1.4
Grade IV	4	0.8	0	0
Bursitits				
Praepatellaris	1	0.2	1	1.4
Infrapatellaris subcutanea	3	0.6	1	1.4
Infrapatellaris profunda	1	0.2	0	0
Baker’s cyst	21	4.1	4	5.5
Hoffaitis	4	0.8	0	0
Quadriceps tendinopathy	3	0.6	1	1.4
Patellar tendinopathy	14	2.8	2	2.7
Articular effusion	123	24.3	2	2.7
Plica thickening				
Plica suprapatellaris	1	0.2	0	0
Plica mediopatellaris	15	3.0	1	1.4
Osteochondritis dissecans	4	0.8	0	0
Morbus Osgood Schlatter	14	2.8	1	1.4
Ganglion cyst	9	1.8	1	1.4
Osteochondroma	5	1.0	2	2.7
Meniscal cyst	6	1.2	0	0
Intra-articular loose body	3	0.6	0	0

**Table 2:** Anatomical parameters.

Anatomical Parameters	Mean	Minimum	Maximum	SD
Tibial tubercle deviation (mm)	10.02	3.00	18.00	2.90
Patella tilt angle (°)	6.97	1.00	34.00	6.20
Bony sulcus angle (°)	140.31	123.00	162.00	10.15
Cartilaginous sulcus angle (°)	145.09	132.00	164.00	5.88
Lateral trochlear inclination (°)	12.97	7.00	24.00	3.36
Insall–Salvati ratio	1.09	0.80	1.41	0.16
Anterior tibial translocation (mm)	3.26	1.00	8.00	1.22

SD = standard deviation.

**Table 3:** Anatomical parameters of the control group.

Anatomical Parameters	Mean	Minimum	Maximum	SD
Tibial tubercle deviation (mm)	9.40	3.00	16.00	2.54
Patella tilt angle (°)	3.97	2.00	17.00	4.10
Bony sulcus angle (°)	136.10	117.00	156.00	8.20
Cartilaginous sulcus angle (°)	139.90	122.00	159.00	4.92
Lateral trochlear inclination (°)	16.34	8.00	28.00	3.35
Insall–Salvati ratio	1.05	0.84	1.30	0.13
Anterior tibial translocation (mm)	4.40	1.00	7.00	1.15

SD = standard deviation.

**Table 4:** Distribution of detected anatomical variants in patients with knee pain and the control group.

Anatomical Situation	Knee Pain Group		Control Group	
	Number of Patients	%	Number of Patients	%
Patella alta	125	24.7	16	21.9
Patella shape				
Wiberg type I	200	39.4	29	39.7
Wiberg type II	187	36.9	25	34.3
Wiberg type III	120	23.7	19	26.0
Tibial tubercle deviation > 15 mm	20	3.9	2	2.7
Patella tilt angle > 5°	199	39.3	8	11.0
Bony sulcus angle > 150°	101	19.9	4	5.5
Cartilaginous sulcus angle > 150°	120	23.7	5	6.8
Lateral trochlear inclination < 11°	125	24.7	7	9.6
Anterior tibial translocation > 7 mm	2	0.4	0	0
Patellar lateralisation	97	19.1	12	16.4

**Table 5:** Results of a binary logistic regression model predicting patellofemoral chondral lesions in the knee pain group.

Variable	*p*-Value	95% Confidence Interval
Patella tilt angle: ≤5° vs. >5°	<0.001	0.019–0.052
Bony sulcus angle: ≤150° vs. >150°	0.002	0.041–0.133
Cartilaginous sulcus angle: ≤150° vs. >150°	0.012	0.028–0.323
Lateral trochlear inclination: <11° vs. ≥11°	<0.001	0.013–0.053
Patella alta: yes vs. no	0.632	0.086–5.324
Anterior tibial translocation: ≤7 mm vs. >7 mm	0.570	0.571–1.362
Patella shape: Wiberg type I vs. type II and type III	0.019	0.018–0.171
Patellar lateralisation: yes vs. no	0.023	0.041–0.343
Tibial tubercle deviation: ≤15 mm vs. >15 mm	0.377	0.273–1.634

**Table 6:** Results of a *t*-test for paired samples in patients with and without patellofemoral chondral lesions of the knee pain group.

Variable	*p*-Value	95% Confidence Interval
Patella tilt angle	0.002	−7.799–(−5.673)
Bony sulcus angle	0.017	−10.491–(−6.991)
Cartilaginous sulcus angle	0.007	−7.388–(−5.368)
Lateral trochlear inclination	<0.001	2.138–3.442
Insall–Salvati Index	0.478	−0.028–3.226
Anterior tibial translocation	0.252	−0.199–2.040
Tibial tubercle deviation	0.091	−0.929–0.178

**Table 7:** Results of a *t*-test for unpaired samples comparing several anatomical parameters of the knee pain group to the control group.

Variable	*p*-Value	95% Confidence Interval
Patella tilt angle	0.021	−5.231–(−1.962)
Bony sulcus angle	0.042	−4.102–(−1.003)
Cartilaginous sulcus angle	0.038	−4.673–(−1.486)
Lateral trochlear inclination	0.014	1.898–2.983
Insall–Salvati Index	0.832	−2.943–4.463
Anterior tibial translocation	0.412	−1.023–3.187
Tibial tubercle deviation	0.234	−1.943–1.243

## Data Availability

The datasets generated during and/or analysed during the current study are available from the corresponding author on reasonable request.

## Abbreviations

Magnetic Resonance Imaging	MRI
For example	e.g.,
Standard deviation	SD
Picture archiving and communication system	PACS
Fat-saturated proton density-weighted turbo spin echo	PD-tse-fs
Repetition time	TR
Echo time	TE
Patellofemoral	PF
Tibiofemoral	TF
